# Heart transplantation in India

**DOI:** 10.1016/j.ihj.2021.06.013

**Published:** 2021-07-06

**Authors:** Gaurang Vaidya

**Affiliations:** Department of Cardiology, University of Kentucky, Lexington, KY, USA

**Keywords:** Heart transplant, India, Global health

As a medical student in a government medical school in Mumbai, I cared for patients with heart failure. I particularly remember a 63-year old man with ischemic cardiomyopathy. After instituting the guideline-directed management of heart failure, I planned for a defibrillator for primary prevention of sudden cardiac death. But the chasm between theory and practice was vast: he declined the defibrillator because the cost (approximately Rs. 3,00,000 or USD 4000) was more than his annual income and he could not understand the value of an intervention that made no difference in how he felt. I don't know what happened to this gentleman after our brief connection years ago, but with the gaps between best medical practice and his care, I doubt he did well.

**Fig. 1:**
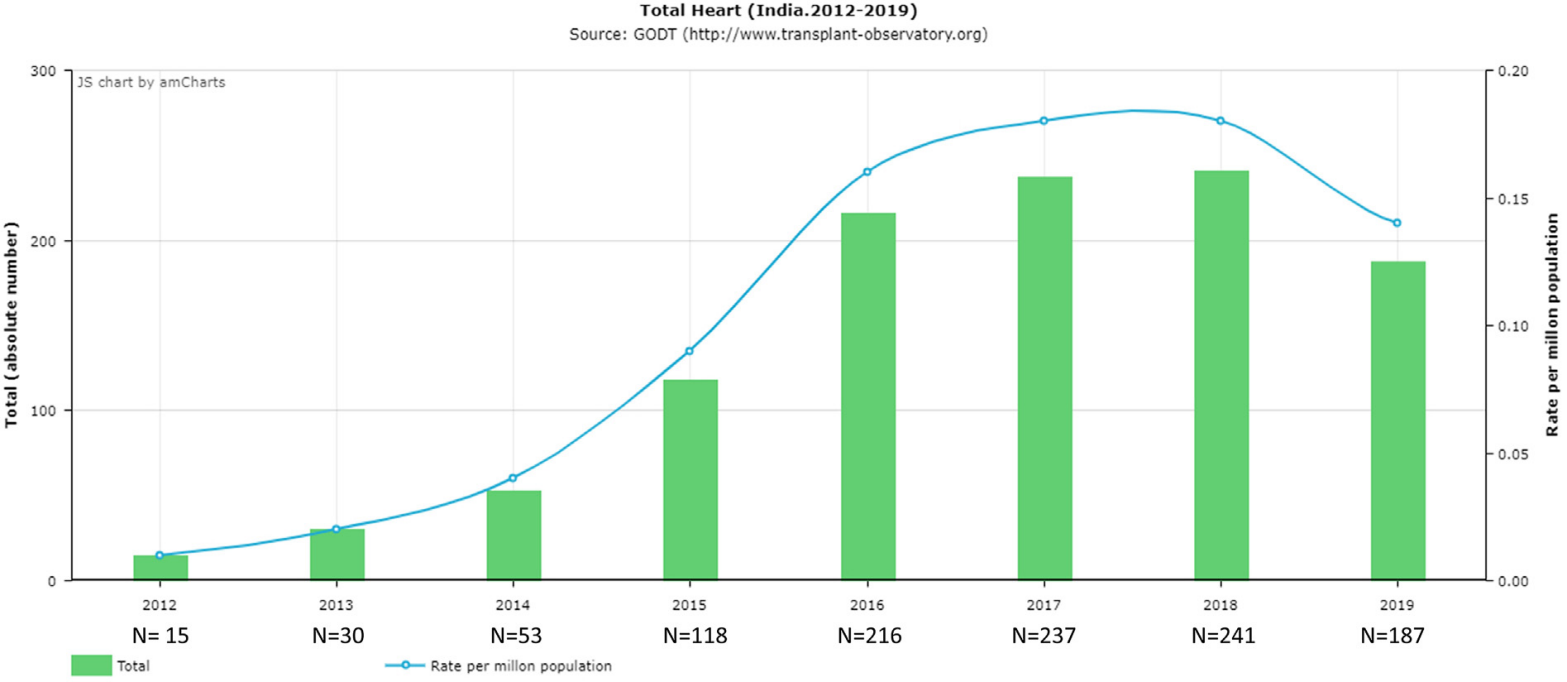
The trend of heart transplants in India from 2012 to 2019 (Source: Global Observatory on Donation and Transplantation).

Much has changed in India over the 8 years since I came to the United States to pursue advanced training. The number of heart transplants has risen exponentially in India in the recent years, from 53 heart transplants in 2014 to 241 transplants in 2018 and 187 in 2019 (Fig. 1).^1^ In fact, in 2018, India had the second highest solid organ transplant volume, after United States, among World Health Organization member countries worldwide.^1^^,^^2^

In November 2019, a local Indian newspaper described how a donor heart was transported 200 miles to a Mumbai hospital in record 80 min, a distance that would take 5 hours by road. The feat required the coordination of multiple air-traffic control units as the helicopter with the donor heart traversed dense air-transport routes. The heart was successfully transplanted in an LVAD patient. This feat exemplifies India's enthusiasm, momentum, and untapped potential of heart failure innovation. Some of the challenges (including financial) may be insurmountable in short term, but others such as lack of health literacy, technology, infrastructure and advanced training among cardiologists can be corrected.

The early history of heart transplantation is well-known: Dr. Christiaan Barnard performed the first heart transplantation in Capetown, South Africa on December 3, 1967. It is less well-known that the fourth surgeon to attempt heart transplant was Dr. Prafulla Sen in Mumbai, India on February 17, 1968.^3^ While the initial interest in heart transplant waned internationally until the discovery of calcineurin inhibitors, heart transplantation in India also stalled due to other barriers, namely the lack of basic healthcare infrastructure. Thus, it was not until 1994 that deceased organ donation was legalized, and the first successful heart transplant was performed.

Recognizing the importance of transplant infrastructure, a 2011 amendment to the Transplantation of Human Organs Act defined the role of transplant coordinators and advisory committees.^2^ This Act established the brain-death declaration team and provided framework for maintenance of a national organ donation registry. In 2014, another amendment clarified the donor and recipient screening practices.^2^ A national registry for donors and recipients was established, with simplification of brain death certification.

The National Organ and Tissue Transplant Organization (NOTTO) was established in 2014 to oversee the organ procurement and distribution. Prior to NOTTO's inception, organ transplantation in India lacked coordination, standardization and national oversight. Illegal organ trade was rife, resulting in multimillion-dollar scandals. Heart transplants were being performed sporadically at certain centers without national dissemination. Among its various functions, NOTTO is entrusted with maintaining the waiting list and post-transplant outcome data, bringing significant organization, legislation and transparency to the organ procurements nationwide. Since its commencement, NOTTO has instituted several policy guidelines and protocols that every transplanting center is expected to follow. The establishment of the Indian Network for Organ Sharing (INOS) in the state of Tamil Nadu improved the organ procurement rates and set a precedence for other states to follow. This has been further coordinated by NOTTO at the national level.

Like any other country, the rate-limiting step in heart transplantation is the scarce supply of donor hearts. But in India, other barriers also exist. Rates of voluntary organ donation are low and approaching grieving relatives remains the major mode of donor identification. This is often fraught with poor awareness, religious misinterpretation or even taboo. Moreover, there is a lack of awareness among treating physicians regarding the availability of advanced heart failure therapies, reducing the referral of eligible patients to appropriate centers. Another highlight is the geographical disparity in donor availability and transplant rates, with transplants predominantly performed in southern India, largely due to religious beliefs and lack of health literacy in North India which preclude organ donation.^4^

In addition to mistrust and taboo, financial barriers are significant. Various hospital websites quote a starting cost from Rs. 20,00,000 (USD 30,000), not including the expensive post-transplant care. In a country where the averageper-capita annual national income is estimated at Rs. 1,30,000 (USD 1800),^5^ heart transplantation can be a financially insurmountable proposition, despite the efforts of the National Organ and Transplant Program (NOTP) to finance transplantation for patients below the poverty line (income < Rs. 30 or $0.50/day). Additional disparity exists as organ allocation may be skewed towards the rich or medical tourists from other countries.^6^

However, India is working to overcome these barriers. In August 2018, India created a world record of over 24,000 organ donation pledges in one day, and then surpassed its own record in November 2019 with more than 54,000 pledges. This is enormous when one considers the previous record, held by United States, was 3200 pledges in one day. The feats were accomplished through awareness campaigns conducted in collaboration with NOTTO and non-governmental organizations (NGO) like the MOHAN foundation (Multi Organ Harvesting Aid Network, https://www.mohanfoundation.org). Such campaigns strive to erase the religious stigma associated with organ donation. In addition, an India's Heart Failure Society was inaugurated in 2006 which evolved into the Indian Society of Heart and Lung Transplantation (inshlt.org.in). The society aims to create an awareness among medical professionals and the public regarding the developments in advanced heart failure therapies in India. In accordance to this goal, the society holds annual and midterm meetings with several hundred attendees. The Indian Heart Journal and the Indian Journal of Thoracic and Cardiovascular Surgery are at the forefront of recognizing the current and the ongoing advances in heart transplantation in India.

Presently, there are over 78 centers in India which perform heart transplants ^4^ and India is the foremost referral center for heart transplants in South Asia.^7^ In addition, organ transport through ‘transport green corridor’ has been given a high priority, with new timing records accomplished with each transplant.

The reported post-transplant survival has been satisfactory. In a single center experience from 2012 to 2019 of 257 adult and pediatric heart transplants, the authors reported a one-year survival of 81%.^8^ During the same time period, the international society of heart and lung transplantation (ISHLT) reported a 1-year survival of 91% among adults.^9^ Combined heart and lung transplantation for pulmonary hypertension in India is still in its nascency but promising outcomes have been reported.^10^ Being a developing country, India faces unique challenges such as unpredictable organ transportation, inconsistent medication supply and limited opportunities for post-transplant follow-up due to multitude of patient and institutional socioeconomic barriers. India still grapples with tropical infections, water-borne illnesses and tuberculosis, all of which can severely affect post-transplant survival independent of the traditional predictors such as ischemic time or hemodialysis.

Waiting-list mortality is similarly high for reasons stated above, in addition to the lack of bridging mechanical circulatory support (MCS) option. Durable ventricular assist device implantation started in India in 2012 but further growth has been hampered by the paucity of financial, infrastructure and political backing.^11^ Availability of viable bridging MCS options is important for several end-stage heart failure patients in India as late presentations or referrals are common. In addition, the absence of insurance reimbursement translates to patients bearing all the expenses. The above issues are in stark contrast to the western countries and addressing these may need overhauling measures which have been hitherto untested or unreported. While 241 heart transplants in 2018 in India is impressive, this constitutes only 5% of the heart transplants reported in the international ISHLT registry in the same year.^9^ For a country with the second largest population in the world, with an estimated prevalence of heart failure in several millions, the transplant rates are still under-whelming. Despite the record organ pledges, the national heart donation rate remained paltry at 0.14 per million population in 2019 compared to >10 per million in the US. ^1^ All the initiatives are focused on adult transplants only, while pediatric organ donations remain atypical.^11^

To sustain the current momentum of organ transplantation in India, further infrastructure is necessary, including recruitment of experienced cardiologists, surgeons, coordinators and infectious disease providers. Despite the rising transplant numbers, the enthusiasm has not been shared by cardiologists in training in India.^12^ The availability of advanced heart failure management has not percolated to the community hospitals where majority of patients seek their healthcare needs. Consequently, the post-transplant care remains a responsibility of a handful of specialized centers in bigger cities. There is a dire need for more institutions offering advanced heart failure management training to cardiologists and nurses. These healthcare professionals are absolutely necessary for an optimal post-transplant survival, which is the yardstick used to measure a nation's performance. Early donor identification and family grief counseling training is needed in smaller hospitals and outreach centers. Transparency in transplant failure rates and institutional accountability is essential. The vast financial burden of building a thriving transplantation scene in India cannot be sustainably borne by the government alone and will need participation of several NGOs and medical insurance companies, apart from a strong political willpower. Expert heart failure cardiologists from western countries can have a lot to contribute to this rising landscape.

When I began training in the United States, I would not have predicted the enormous advances in India in less than a decade. I recently cared for a 58-year old man in the United States with cardiogenic shock complicated by refractory VT. He required a percutaneous left ventricular assist device and later extracorporeal membrane oxygenation support to stabilize him to successful heart transplantation. The barriers I had experienced when caring for my heart failure patient in India did not exist: there was no concern for expense for this insured patient and he had full faith in the medical system. As he presents for routine clinic appointments, one would never guess that weeks earlier he was near death. I see him in clinic, but I think of my patient, years earlier in India. When I cared for him then, I was sure he would die of end-stage heart failure, not due to lack of effective therapies, but due to his inability to receive them. But thanks to improvements in infrastructure and awareness, I look forward to a future in India where a patient could present with end-stage heart failure and experience an outcome as joyous and miraculous.
